# Greater lung cancer polygenic risk score in higher air pollution areas linked to greater rate of lung adenocarcinoma: a single-centre study in East Asia

**DOI:** 10.1136/bmjresp-2024-002899

**Published:** 2025-10-13

**Authors:** Szu-Ling Chang, Peng-Min Chuan, Chih-Hung Lai, Hui-Wen Yang, Yi-Ming Chen, Han-Shui Hsu, I-Chieh Chen

**Affiliations:** 1 Department of Anesthesiology, Taichung Veterans General Hospital, Taichung, Taiwan; 2 School of Medicine, National Yang-Ming Chiao Tung University, Taipei, Taiwan; 3 Institute of Emergency and Critical Care Medicine, National Yang Ming Chiao Tung University School of Medicine, Taipei, Taiwan; 4 Department of Medicine and Cardiovascular Center, Taichung Veterans General Hospital, Taichung, Taiwan; 5 Department of Medical Research, Taichung Veterans General Hospital, Taichung, Taiwan; 6 Graduate Institute of Clinical Medicine, College of Medicine, National Chung-Hsing University, Taichung, Taiwan; 7 Division of Allergy, Immunology and Rheumatology, Taichung Veterans General Hospital, Taichung, Taiwan; 8 Department of Post-Baccalaureate Medicine, College of Medicine, National Chung-Hsing University, Taichung, Taiwan; 9 Precision Medicine Research Center, College of Medicine, National Chung-Hsing University, Taichung, Taiwan; 10 Division of Thoracic Surgery, Department of Surgery, Taipei Veterans General Hospital, Taipei, Taiwan; 11 Master Program in Precision Health, National Chung Hsing University, Taichung, Taiwan

**Keywords:** Lung Cancer, Clinical Epidemiology

## Abstract

**Background:**

Lung cancer, a leading cause of cancer deaths, is influenced by smoking, air pollution and genetic factors. This study investigated the association between lung cancer polygenic risk score (PRS) and air pollution in lung adenocarcinoma (LUAD) cases in East Asia.

**Methods:**

This Taiwanese case-control study included 57 257 participants, of whom 1059 were diagnosed with lung cancer and 857 had LUAD. Excluding individuals with missing PRS data, the final study group comprised 648 LUAD patients and 6480 age- and gender-matched controls. Logistic regression models were employed to assess the association between PRS and LUAD risk, and interaction effects between PRS and particulate matter (PM2.5/PM10) exposure were evaluated.

**Results:**

PGS000070 demonstrated an OR of 2.796 (95% CI 2.236 to 3.497, p<0.001), while PGS000392 exhibited an OR of 1.938 (95% CI 1.559 to 2.409, p<0.001). Higher PM exposure increased LUAD risk among individuals in the highest quartile (Q4) of both PRSs compared with the lowest quartile (Q1). In the smoking subgroup, individuals in Q4 for PGS000070 showed significantly heightened LUAD risk when exposed to higher PM2.5 and PM10 levels, with ORs of 4.08 (p<0.0001) and 2.897 (p<0.0001), respectively. However, the interaction effect of PRS (PGS000070 and PGS000392) and PM exposure on LUAD risk was not statistically significant.

**Conclusion:**

This hospital-based study indicated that LUAD patients had higher PRSs and greater exposure to PM. However, the interaction effect between PRS (PGS000070 and PGS000392) and PM exposure on LUAD risk was not statistically significant, suggesting these factors act independently. The accumulation of air pollution did not show a gradual increase in LUAD risk. Notably, the association between PRS and air pollution was more pronounced in the smoking subgroup for PGS000070 but not for PGS000392.

**WHAT IS ALREADY KNOWN ON THIS TOPIC**

- Lung cancer, especially lung adenocarcinoma (LUAD), is influenced by smoking, air pollution and genetic factors. Polygenic risk scores (PRSs) are used to evaluate genetic susceptibility to lung cancer; however, the interaction between these factors in East Asian populations remains incompletely understood.

**WHAT THIS STUDY ADDS**

- This study quantifies the link between high PRS scores and increased LUAD risk with elevated PM2.5/PM10 exposure, particularly in smokers. It shows that LUAD patients had higher PRS and PM exposure, but the risk increase was not linear. A stronger PRS-air pollution association was found in smokers for PRS PGS000070.

**HOW THIS STUDY MIGHT AFFECT RESEARCH, PRACTICE OR POLICY**

- This study shows that higher particulate matter exposure is associated with increased LUAD risk in individuals with elevated PRSs. The association patterns differ by PRS and smoking status, highlighting the importance of considering both genetic susceptibility and environmental exposures. These findings highlight the need for improving air quality and promoting smoking cessation to reduce lung cancer risk, particularly in genetically susceptible populations.

## Introduction

 Lung cancer is a leading cause of cancer-related mortality in the current era.^1^ One of the well-established environmental risk factors is smoking.^2^ Cigarette smoking may increase the genetic instability and mutation that induce lung carcinogenesis.^3 4^ In addition, numerous studies have shown that exposure to air pollutants increased the risk of developing lung cancer. Air pollution plays a vital role in lung cancer in never-smokers (LCINS); it has unique clinical and molecular features compared with those in smokers. It was reported to promote inflammatory change in the pre-existing mutated clones to expand in a recent study.^5^

Although environmental risk factors contribute significantly, genetic variants can account for 12%–21% of lung cancer heritability.^6^ Lung adenocarcinomas (LUAD) with epidermal growth factor receptor (EGFR) mutations are common in LCINS, with higher prevalence in females and individuals of East Asian ancestry.^7^ In this area, fine particulate matter ≤2.5 µm (PM2.5) particle is a major contributor to the growing burden of lung cancer deaths.^8^ In the same environmental exposure, some people get lung cancer, but others do not. Some are more susceptible to developing lung cancer due to their genetic mutations, like TP53 tumour-suppressor and EGFR variants.^9^ The genetic-environmental interaction was shown to be additive in the UK Biobank (UKB).^10^

In the past decade, genome-wide association studies (GWASs) have identified 45 risk loci for lung cancer across different ethnic populations.^11^ Recently, the concept of polygenic risk score (PRS) has emerged, representing a composite score that incorporates the presence or absence of multiple genetic variants associated with particular diseases to discern populations at high risk of complex diseases.^12 13^ A recent research identified a link between PRS and the risk of LUAD in women who had never smoked, with the risk increasing along with the level of their exposure to environmental tobacco smoke.^14^

This study aims to explore the genetic factors, specifically PRSs, influencing individual susceptibility to LUAD and to evaluate the association between PM exposure and the risk of LUAD in East Asian groups.

## Materials and methods

### Data source

This retrospective case-control study included 57 257 Taiwanese participants from the Taiwan Precision Medicine Initiative (TPMI) between June 2019 and November 2022.^13^ Data were obtained from Taichung Veterans General Hospital (TCVGH) electronic health records and biospecimens. Demographics, diagnoses, surgical procedures and medication data were collected, and genotyping was performed using the Affymetrix Genome-Wide Taiwan Biobank V.2 (TWB 2.0) single nucleotide polymorphism (SNP) Array.^15^ All participants provided written informed consent in accordance with the Declaration of Helsinki, and the study was approved by the TCVGH IRB (CE24219B).^16^

### Participants

This study analysed data from 57 257 participants in the TCVGH database. Out of all the participants, 1059 patients were clinically diagnosed with lung cancer, confirmed by the International Classification of Diseases, Clinical Modification (ICD-9-CM) diagnosis codes 162.3–162.9, which were recorded either at least twice during outpatient visits or once during hospitalisation. Out of all participants, 857 were clinically diagnosed with LUAD, 26 had small cell carcinoma, 83 had squamous cell carcinoma and 93 had other types of lung cancer (online supplemental table 1). To ensure data accuracy, 209 participants with missing genotyping and air pollution exposure data, as well as a follow-up time of <1 year, were excluded from the analysis. Finally, a total of 648 patients diagnosed with LUAD were included. The index date was defined as the date of lung cancer diagnosis, and patients were followed until the end of the available follow-up period, which extended from January 2000 to December 2021. Age- and gender-matched controls were selected from the TPMI registry using the MatchIt package in R V.4.1.0 with nearest neighbour matching. Each LUAD case was matched to 10 controls of the same sex and similar age (1:10 matching ratio), resulting in a total of 6480 controls.

### Clinical parameters

Patients diagnosed with lung cancer were identified based on ICD-9-CM codes 162.3–162.9. The genetic profile was correlated with clinical parameters, such as age at onset, sex, family history of lung cancer, history of smoking and clinical stages. Cigarette smoking, alcohol drinking and betel nut chewing were ascertained through self-reports of the upper aerodigestive tract cancer patients and defined as ‘yes’ or ‘no’.^13^

### Measurements

This study focused on PM2.5 and PM10 data from 77 Environmental Protection Administration (EPA) air quality monitoring stations across Taiwan.^17^ The stations are classified into six types (general, industrial, traffic, background, national park and rural).^18^ Daily 24-hour average concentrations were used. Participants’ addresses from TPMI were geocoded using Google Maps API^19^ and Taiwan Geospatial One-Stop^20^ to obtain coordinates, and exposure was assigned based on the nearest monitoring station. Air quality data from 2000 to 2021 were retrieved from the EPA open platform. In our study, the 1-, 3-, 5- and 10-year exposure metrics for PM2.5 and PM10 represent the cumulative average daily concentrations over the 12, 36, 60 and 120 months, respectively, prior to each participant’s index date. For each participant, we linked the nearest EPA monitoring station, retrieved daily 24-hour average concentrations, and then calculated the mean exposure over the respective time window (eg, for the 3-year window, all daily values during the 36 months before the index date were averaged).

### Genotyping and quality control

At TCVGH, DNA extraction was automated, and genotyping for each participant was performed using the TWBV.2 customised SNP array (Thermo Fisher Scientific, Santa Clara, California, USA). This array, specifically designed for GWASs with a focus on known risk alleles, included 714 431 SNPs, as previously reported by Wei *et al.*^21^

Genotype calling was centrally conducted at Academia Sinica in batches of approximately 3000 samples to ensure consistency and reduce batch effects. Quality control (QC) was applied at both the sample and SNP levels. Samples with a call rate <95% were excluded, and SNPs were removed if they were monomorphic, had a call rate <95%, a minor allele frequency <0.01 or deviated significantly from Hardy–Weinberg equilibrium (p<1×10⁻⁴).

Following QC, genotype imputation for autosomal chromosomes was performed using the Michigan Imputation Server with the ‘minimac4’ algorithm.^22^ Strand-aligned genotype data were imputed against the 1000 Genomes Project Phase 3 (V.5) reference panel,^23^ and variants with an imputation quality score (INFO) ≥0.3 were retained for downstream analyses.

### Polygenic risk score calculation

The PRSs of the case group and control group were calculated and divided into quartiles. The ORs compare patients in different quartiles of PRS, assessing the actual risk differences for LUAD. In this study, we used the PGS000070^6^ and PGS000392,^24^ which were derived from a discovery analysis of variants associated with lung cancer identified from a Trans-ancestry GWAS for each of 19 curated traits across two ancestry groups from the China Kadoorie Biobank (CKB) and nine ancestry groups from the UKB, respectively. We obtained the list of SNPs and their corresponding effect sizes from the polygenic score catalogue.^25^ PRS was calculated using the ‘score’ function from PLINK V.1.9,^26^ an open-source tool for genetic data analysis, to aggregate the effects of multiple genetic variants weighted by their effect size from the polygenic score catalogue.^13^ Because allele frequency and linkage disequilibrium patterns vary across populations and may affect raw PRS distributions,^27^ we adjusted the scores using the top 10 genetic principal components derived from genome-wide data with PLINK 1.9. Given the high genetic homogeneity of our cohort (>95% Han Chinese ancestry), the risk of residual population stratification was considered minimal.^28^

### Statistical analysis

The sample was merged with air pollution exposure data by unique participants’ scrambled identification number and year of visit. Normality tests were performed using the Shapiro–Wilk test to determine the data distribution. The PRS was treated as a categorical variable and stratified into four groups, based on the deciles of PRS values.^16^ The number of individuals in each group was the same. In this study, participants were grouped into Q1 (0%–25%), Q2 (26%–50%), Q3 (51%–75%) and Q4 (76%–100%) of scores according to these thresholds. Descriptive statistics for continuous variables were presented as mean±SD, and group differences were assessed using student’s t-test. Categorical variables were expressed as number (percent), and differences among groups were evaluated using the χ^2^ test. Univariate logistic regression was used to examine the association between air pollution exposures, PRS and LUAD, adjusting for potential confounders. Two-tailed statistical tests were employed, with significance considered at p<0.05.

Additive^29 30^ and multiplicative^29^ interaction analyses were conducted to assess the joint effect of PRSs and air pollution on LUAD risk. Additive interactions were quantified using three measures: the relative excess risk due to interaction (RERI), the attributable proportion due to interaction (AP) and the synergy index (SI). The 95% CIs for these interaction measures were calculated based on the table provided by T. Anderson.^16 31^ An interaction was considered absent if the 95% CI of RERI or AP included 0 or if the 95% CI of SI included 1.

All analyses were carried out using IBM SPSS Statistics (version 25; IBM Corporation, Armonk, New York, USA) and Statistical Analysis System (SAS, version 9.4; SAS Institute Inc., Cary, North Carolina, USA).

## Results

**Figure 1:**
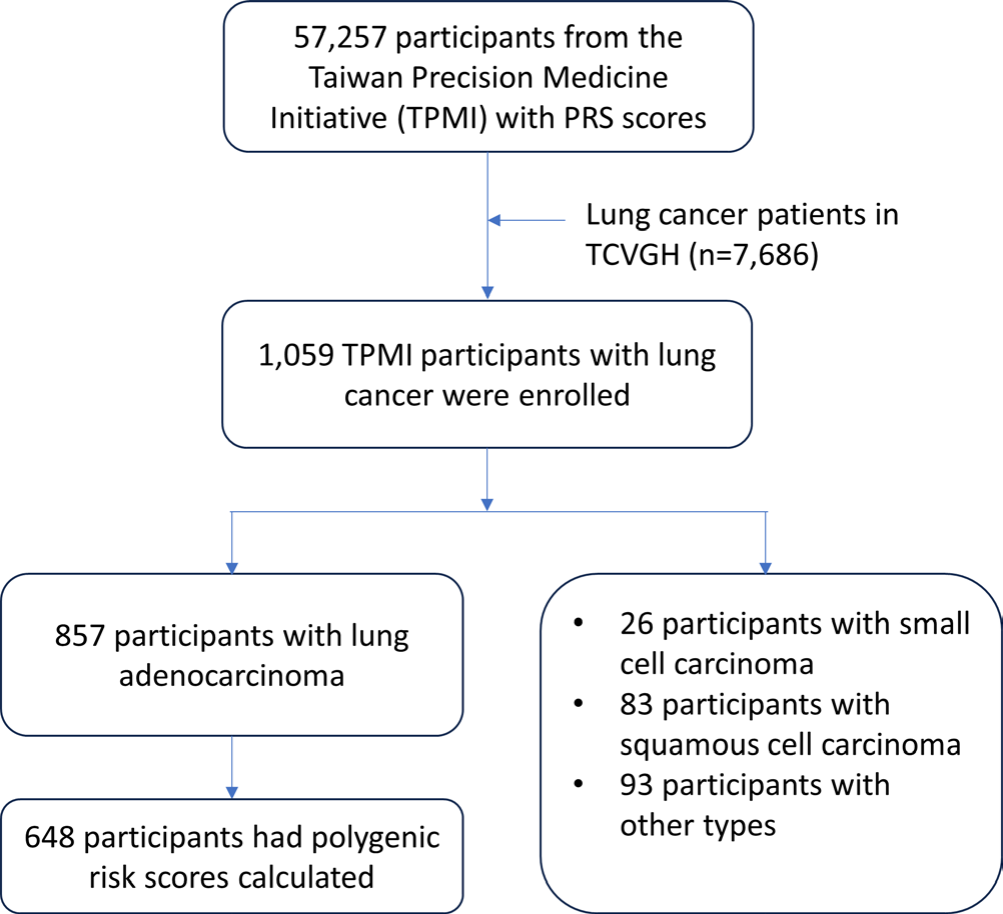
Flow chart for enrolled participants in the study. PRS, polygenic risk score; TCVGH, Taichung Veterans General Hospital.

This study analysed data from 57 257 participants in the TCVGH database, with 2295 excluded for missing data, leaving 54 962 participants. Among them, 648 patients clinically diagnosed with LUAD were enrolled (figure 1). Logistic regression models were used to examine the association between PRSs and LUAD, adjusting for age and gender (online supplemental table 1). Individuals in the highest PRS quartile (Q4) had significantly higher lung cancer risk than those in the lowest quartile (Q1); for example, PGS000070: OR=2.796 (95% CI 2.236 to 3.497, p<0.001) and PGS000392: OR=1.938 (95% CI 1.559 to 2.409, p<0.001). Characteristics of the 648 LUAD patients are shown in table 1. Cases and controls were matched at a 1:10 ratio by age and gender, and the mean age of the cases was 67.16±11.13 years. Both PGS000070 and PGS000392 were significantly higher in cases (p<0.0001). PM2.5 and PM10 exposure levels were also significantly higher in cases across 1-, 3-, 5- and 10-year windows (p<0.0001). Table 2 shows the associations between PRS quartiles, air pollutant exposure and LUAD risk. Higher PM2.5 exposure was significantly associated with increased LUAD risk. Participants in Q4 of PGS000070 had ORs of 2.938–3.224 versus Q1 (p<0.0001), and those in Q4 of PGS000392 had ORs of 1.897–2.018 (p<0.0001). For PGS000070, Q4 remained significantly associated with LUAD regardless of PM10 levels, whereas PGS000392 showed a significant association only under higher PM10 exposure (ORs 1.914–2.01, p<0.0001). Then, we examined PM exposure levels stratified by smoking status and LUAD status. As shown in online supplemental figure 1 and online supplemental table 2, both PM2.5 and PM10 concentrations were higher in LUAD cases compared with controls across 1-, 3-, 5- and 10-year exposure windows, and this pattern was consistent in both never-smokers and ever-smokers. In the control group, statistically significant differences in PM levels were observed between never-smokers and smokers, whereas in the LUAD group, the differences by smoking status were not statistically significant. Overall, LUAD cases consistently exhibited higher PM exposure than controls.

**Table 1:** Characteristics of the study subjects (n=648)

Variables	Case (n=648)		Control (n=6480)		P value
	N	%	N	%	
Sex (n, %)*					1
Female	392	60.49	3920	60.49	
Male	256	39.51	2560	39.51	
Age (mean, SD)	67.16	11.13	67.16	11.12	1
Polygenic risk score (mean, SD)†					
PGS000070	−0.91	0.35	−1.05	0.34	<0.0001
PGS000392	−0.35	0.32	−0.42	0.33	<0.0001
Smoke (n, %)					0.0008
no	458	70.68	4944	76.56	
yes	190	29.32	1514	23.44	
Air pollution (mean, SD)†					
PM2.5					
1 year before index date	24.04	5.59	15.7	2.74	<0.0001
3 years before index date	26.00	5.1	19.54	2.73	<0.0001
5 years before index date	27.57	4.6	21.66	2.79	<0.0001
10 years before index date	30.38	3.87	26.18	2.59	<0.0001
PM10					
1 year before index date	46.74	6.68	37.66	2.61	<0.0001
3 years before index date	49.57	5.61	41.13	2.59	<0.0001
5 years before index date	51.64	4.59	44.56	2.47	<0.0001
10 years before index date	54.09	3.32	50.23	2.46	<0.0001

* Categorical variables were expressed as numbers (per cent) and were analysed using the χ2 test.

† Continuous variables were expressed as mean± SD and were analysed using student’s t-test for normal data distributions.

PM, particulate matter.

**Table 2:** Risk of LUAD in patients exposed to various particulate matters

Variables	PM2.5 <median			PM2.5 ≥median			PM10 <median			PM10 ≥median		
	OR	95% CI	P value	OR	95% CI	P value	OR	95% CI	P value	OR	95% CI	P value
**PGS000070**												
1 year												
Q1	–	–	–	–	–	–	–	–	–	–	–	–
Q4	1.240	0.511 to 3.012	0.6347	3.224	2.471 to 4.208	<0.0001	2.675	1.224 to 5.846	0.0136	2.842	2.175 to 3.715	<0.0001
3 years												
Q1	–	–	–	–	–	–	–	–	–	–	–	–
Q4	1.329	0.6 to 2.946	0.483	3.219	2.462 to 4.209	<0.0001	2.265	1.019 to 5.035	0.0448	2.987	2.287 to 3.903	<0.0001
5 years												
Q1	–	–	–	–	–	–	–	–	–	–	–	–
Q4	1.752	0.797 to 3.849	0.163	3.183	2.435 to 4.159	<0.0001	2.513	1.188 to 5.316	0.016	2.860	2.186 to 3.74	<0.0001
10 years												
Q1	–	–	–	–	–	–	–	–	–	–	–	–
Q4	2.771	1.483 to 5.175	0.0014	2.938	2.234 to 3.865	<0.0001	2.565	1.490 to 4.416	0.0007	3.024	2.283 to 4.007	<0.0001
**PGS000392**												
1 year												
Q1	–	–	–	–	–	–	–	–	–	–	–	–
Q4	1.182	0.453 to 3.081	0.7325	2.018	1.559 to 2.612	<0.0001	1.679	0.723 to 3.901	0.2281	1.96	1.513 to 2.54	<0.0001
3 years												
Q1	–	–	–	–	–	–	–	–	–	–	–	–
Q4	1.630	0.663 to 4.008	0.2872	1.925	1.487 to 2.492	<0.0001	1.710	0.705 to 4.145	0.2354	2.010	1.553 to 2.602	<0.0001
5 years												
Q1	–	–	–	–	–	–	–	–	–	–	–	–
Q4	1.994	0.841 to 4.727	0.1173	1.952	1.507 to 2.529	<0.0001	1.658	0.741 to 3.713	0.219	1.914	1.476 to 2.482	<0.0001
10 years												
Q1	–	–	–	–	–	–	–	–	–	–	–	–
Q4	2.039	1.081 to 3.844	0.0277	1.897	1.454 to 2.476	<0.0001	1.665	0.996 to 2.783	0.0517	1.985	1.506 to 2.617	<0.0001

ORs were estimated using univariable logistic regression models, adjusted for age and gender.

LUAD, lung adenocarcinoma; PM, particulate matter.

To further clarify the role of smoking, we evaluated its association with LUAD risk in combination with PRSs (PGS000070 and PGS000392) and air pollution. As shown in online supplemental figure 2, LUAD patients generally exhibited higher PRS values compared with controls, regardless of smoking status. Moreover, online supplemental table 3 shows that cigarette smoking was associated with an increased risk of LUAD, with smokers exhibiting a 1.391-fold higher risk compared with non-smokers when considering their PRSs (PGS000070). Additionally, smokers demonstrate a 1.461-fold higher risk of LUAD compared with non-smokers, based on their PRSs PGS000392. Then, we conducted a stratified analysis using logistic regression to estimate the association between PRSs and air pollution with the risk of LUAD, stratified by smoking and non-smoking status. As shown in table 3, in the smoking group, compared with the lowest quartile (Q1) of PGS000070, the risk significantly increased when individuals were exposed to higher PM2.5 levels and PM10 (above the median), with ORs of 4.08 (p<0.0001) and 2.897 (p<0.0001), respectively, in the highest quartile (Q4) of PGS000070. In contrast, the ORs of LUAD were 3.01 and 2.85 (p<0.0001) of higher PM2.5 levels and PM10, respectively, in the non-smoking group.

**Table 3:** Risk of LUAD in patients who smoke and are exposed to various particulate matters (PGS000070)

Variables	Smoking			Smoking		
	PM2.5 <median			PM2.5 ≥median		
	OR	95% CI	P value	OR	95% CI	P value
**PGS000070**						
Q1	–	–	–	–	–	–
Q4	0.881	0.256 to 3.036	0.8415	4.080	2.409 to 6.912	<0.0001

	Non-smoking				Non-smoking			
	PM2.5 <median				PM2.5 ≥median			
Q1	–	–	–	–	–	–	–	–
Q4	2.169	0.539	8.724	0.2757	3.010	2.207	4.106	<0.0001

	Smoking			Smoking		
	PM10 <median			PM10 ≥median		
	OR	95% CI	P value	OR	95% CI	P value
Q1	–	–	–	–	–	–
Q4	3.003	0.784 to 11.498	0.1085	2.897	1.739 to 4.827	<0.0001

	Non-smoking				Non-smoking			
	PM10 <median				PM10 ≥median			
Q1	–	–	–	–	–	–	–	–
Q4	2.585	0.986	6.779	0.0536	2.850	2.076	3.913	<0.0001

ORs were estimated using univariable logistic regression models, adjusted for age and gender.

LUAD, lung adenocarcinoma; PM, particulate matter.

In table 4, only in the non-smoking group, compared with Q1, the risk significantly increased when individuals were exposed to higher PM2.5 levels and PM10 (above the median), with ORs of 2.199 (p<0.0001) and 2.183 (p<0.0001), respectively, in Q4 of PGS000392.

**Table 4:** Risk of LUAD in patients who smoke and are exposed to various particulate matters (PGS000392)

Variables	Smoking			Smoking		
	PM2.5 <median			PM2.5≥median		
	OR	95% CI	P value	OR	95% CI	P value
**PGS000392**						
Q1	–	–	–	–	–	–
Q4	1.345	0.289 to 6.254	0.705	1.564	0.978 to 2.501	0.062

	Non-smoking				Non-smoking			
	PM2.5 <median				PM2.5 ≥median			
	–	–	–	–	–	–	–	–
	1.081	0.311	3.753	0.9026	2.199	1.610	3.003	<0.0001

	Smoking			Smoking		
	PM10 <median			PM10 ≥median		
	OR	95% CI	P value	OR	95% CI	P value
Q1	–	–	–	–	–	–
Q4	2.922	0.577 to 14.799	0.195	1.470	0.920 to 2.348	0.107

	Non-smoking				Non-smoking			
	PM10 <median				PM10 ≥median			
Q1	–	–	–	–	–	–	–	–
Q4	1.248	0.450	3.461	0.6708	2.183	1.593	2.991	<0.0001

ORs were estimated using univariable logistic regression models, adjusted for age and gender.

LUAD, lung adenocarcinoma; PM, particulate matter.

We further examined the joint effects of PRSs (PGS000070 and PGS000392), smoking status and PM2.5 and PM10 exposures on lung cancer risk (online supplemental tables 4 and 5). For PGS000070, significant associations were observed across all smoking categories, but only under higher PM exposure conditions. Among current smokers, individuals in the highest PRS quartile (Q4) exhibited markedly elevated risks when exposed to PM2.5 ≥median (OR=5.625, 95% CI 1.70 to 18.61, p=0.0047) and PM10 ≥median (OR=5.33, 95% CI 1.63 to 17.49, p=0.0058), whereas no associations were detected at lower exposure levels. In former smokers, Q4 individuals showed significantly increased risks with PM2.5 ≥median (OR=3.672, 95% CI 2.00 to 6.73, p<0.0001) and PM10 ≥median (OR=2.358, 95% CI 1.31 to 4.26, p=0.0045), but not with lower exposures. Among never-smokers, elevated genetic risk was consistently associated with lung cancer susceptibility when combined with higher particulate exposure (PM2.5 ≥median: OR=3.01, 95% CI 2.21 to 4.11, p<0.0001; and PM10 ≥median: OR=2.85, 95% CI 2.08 to 3.91, p<0.0001). No significant associations were observed among never-smokers exposed to lower PM levels. For PGS000392, distinct patterns were observed. No statistically significant associations emerged among current or former smokers across either PM2.5 or PM10 strata (all p>0.1). In contrast, strong associations were found among never-smokers exposed to higher particulate concentrations. Specifically, Q4 individuals had significantly elevated risks with PM2.5 ≥median (OR=2.199, 95% CI 1.61 to 3.00, p<0.0001) and PM10 ≥median (OR=2.183, 95% CI 1.59 to 2.99, p<0.0001), but no associations were evident under lower exposure conditions. These findings indicate that genetic susceptibility, as captured by PRSs, interacts with both smoking status and air pollution exposure. While PGS000070 revealed gene–environment interactions across all smoking categories under high PM conditions, PGS000392 demonstrated a more specific effect among never-smokers exposed to elevated particulate levels.

Next, we examined the interaction effect of PRS and PM2.5/PM10 on LUAD risk by calculating the additive (AP, RERI and SI) and multiplicative interactions. As shown in online supplemental table 6, the interaction effect between PRS (PGS000070 and PGS000392) and PM exposure on LUAD risk was not statistically significant. Specifically, the 95% CIs for RERI and AP included 0, and the 95% CIs for the SI included one for both PRSs, indicating no evidence of additive or multiplicative interactions. Our results suggest that the combined effect of these two factors on LUAD risk is not greater than the sum of their individual effects.

Additionally, the investigation focused on the association between PRSs, PM exposure and clinical staging, stratifying LUAD into early stages (stage I+stage II) and late stages (stage III+IV) as illustrated in online supplemental figure 3. Among those exposed to higher PM10 levels (above the median), individuals in the lowest quartile (Q1) of both PGS000070 (online supplemental figure 3A) and PGS000392 (online supplemental figure 3B) exhibited a higher percentage of late-stage LUAD. Similarly, when individuals were exposed to higher PM2.5 levels (above the median), individuals in the lowest quartile (Q1) of PGS000070 also exhibited a higher percentage of late-stage LUAD. However, these subgroup analyses involved relatively small sample sizes, and the findings should therefore be interpreted with caution.

## Discussion

In this retrospective case-control study, the patients with LUAD had higher PRS quartile and higher particulate matter (PM) exposure. However, cumulative PM exposure over 1-, 3-, 5- and 10-year windows did not show a clear gradual increase in LUAD risk, which may be influenced by the use of a single residential address and the lack of longitudinal residential history. The association between PRS and air pollution was found to be increased in the smoking group in PGS00070, but no such increase was observed in PGS000392.

In the case group, average PM exposures over 1-, 3-, 5- and 10-year periods were higher than in the control group, with the mean values increasing over longer time windows. When stratified by PRS (PGS000070), the ORs ranged from 2.938 to 3.224, but no consistent year-by-year increase in risk was observed. The finding is also similar in PGS000392, with the OR increased from 1.897 to 2.018. The research finding elaborates on the additive interaction of genetic variation and air pollution on LUAD. Similar results were reported in the UKB, where those with high genetic risk patients under long-term exposure to air pollution increased lung cancer risk.^10 32^ In the previous study, the air pollution was a single measurement at baseline.

Unlike previous studies that measured air pollution only once at baseline, our study used continuous data up to 10 years prior to the index date, allowing a more accurate assessment of long-term PM exposure. Exposure was categorised into 1-, 3-, 5- and 10-year windows to evaluate its association with LUAD, a cancer type less related to smoking. Given the higher air pollution levels in East Asia compared with the UK,^33^ this may partly explain the elevated lung cancer risk observed relative to UKB findings.

The WHO has heightened the risk of lung cancer, with an estimated 250 000 deaths worldwide each year attributed to air pollution.^34^ Air pollution may promote lung cancer through inflammation, DNA damage and epigenetic changes, with microorganisms carried by PM potentially accelerating this process.^35^ Hill *et al* reported elevated interleukin-1 beta (IL-1β) expression during PM2.5-induced lung inflammation.^5^ Mutated cells may remain dormant until triggered by exposures such as smoke or air pollutants, leading to early tumour development.^36^ Our findings are consistent with previous epidemiological and molecular studies.

When accounting for smoking, individuals in Q4 of PGS000070 with high PM2.5 exposure had the highest LUAD risk (OR 4.08), which was slightly lower in non-smokers (OR 3.01). In contrast, PGS000392 showed no increased risk overall but a higher risk in non-smokers (OR 2.199). This discrepancy may reflect the characteristics of LUAD in East Asia, where it is often seen in never-smokers, predominantly females, and is associated with EGFR mutations.^37^ Moreover, the two PRSs were derived from different discovery cohorts: PGS000070 was developed using the CKB, predominantly East Asian, whereas PGS000392 was based on the UKB, primarily of European ancestry. Differences in the training populations may underlie the observed heterogeneity in associations. In our Taiwanese cohort, PGS000392 showed a significant association with LUAD risk mainly in non-smoking individuals, whereas PGS000070 exhibited consistent associations across both smoking and non-smoking subgroups. These discrepancies likely reflect differences in allele frequencies, linkage disequilibrium patterns and gene–environment interactions between populations, highlighting the importance of ancestry-specific PRS for accurate risk prediction.

In our interaction effect analysis, no significant multiplicative or additive interaction between PRS (PGS000070 and PGS000392) and PM2.5/PM10 exposure for LUAD was observed. This suggests that the effects of these two factors on LUAD onset are independent of each other, and the combined effect of PRS and PM exposure on LUAD risk does not exceed the sum of their individual effects. However, it should be noted that both higher PRS and greater PM exposure are independently associated with increased LUAD risk.

This study also observed that individuals in the lowest PRS quartile with high PM exposure presented with a higher proportion of late-stage lung cancer at diagnosis. Although the mechanisms underlying this pattern are not yet clear, several possible explanations may be proposed. First, individuals with lower genetic susceptibility may have fewer early warning symptoms or slower subclinical progression, leading to delayed detection. Second, such individuals may perceive themselves at lower risk and thus be less engaged in preventive healthcare or medical surveillance, contributing to later-stage diagnoses. Third, unmeasured factors such as occupational exposures, healthcare access and screening practices may confound this association. Finally, this observation may in part reflect random variation due to smaller subgroup sizes. These findings highlight the need for future studies incorporating longitudinal smoking history, detailed clinical follow-up and broader environmental and behavioural factors to clarify the interaction between genetic risk, air pollution exposure and lung cancer stage at presentation.

In our study, PRS takes negative values, which may raise questions regarding interpretation. It is important to note that the sign of the PRS does not indicate low risk or ‘negative’ disease status; rather, it reflects the relative balance of risk and protective alleles compared with a reference population. Although PRS values ranged from negative to positive, the mean PRS for both PGS000070 and PGS000392 was significantly higher in cases than in controls (PGS000070: −0.91 vs −1.05; PGS000392: −0.35 vs −0.42), indicating higher genetic susceptibility among cases. Regarding case classification, we relied on both ICD-9-CM codes 162.3–162.9 and a lung cancer registry list to ensure accurate identification. While some cases were excluded due to incomplete data, this is unlikely to systematically bias PRS values. These observations highlight that variability in PRS is expected at the individual level, and meaningful distinctions are most reliably interpreted at the group level.

Strengths of our study included the use of a large sample size in East Asia and the assessment of long-term air pollution exposures, estimated as cumulative mean concentrations over time windows of up to 10 years. However, the current study has several limitations. First, we used the air quality index numbers corresponding to the patient’s address provided at the time of enrolment. However, we cannot ascertain whether the addresses provided by participants accurately reflected their actual residential locations. Moreover, the estimated level of air pollution exposure, based on registered residential addresses, may not fully capture an individual’s true daily exposure to air pollutants over the extended observation period. In particular, unmeasured residential mobility during the observation period may have affected the accuracy of long-term exposure assessment. Satellite-based PM data were not available for this study; exposure was estimated from the nearest ground monitoring stations, which may not fully capture individual mobility. Integration with satellite-derived data will be considered in future research to improve exposure assessment. Second, the collection of clinical data from hospitals presented significant challenges. Limited by our resources, participation was confined to a single hospital centre for this study. Consequently, there may be a potential for sampling bias. Future research should aim to use a population-based dataset to mitigate this issue. Third, other air pollution such as home tobacco use, cooking oil fumes and occupational pollution was not counted into consideration, which could lead to potential residual confounding. Fourth, information on smoking status was only available at the time of TPMI enrolment, and we were unable to distinguish between current and former smokers or evaluate the duration of smoking cessation. This limitation may have affected the assessment of gene–environment interactions, given that prior evidence suggests differential PM effects by smoking status. Fifth, this retrospective case-control design limited our ability to fully account for confounding factors. Moreover, some subgroup analyses, particularly those stratified by smoking status and cancer stage, involved small sample sizes, reducing statistical power. Thus, these findings should be interpreted with caution. Future cohort studies with detailed smoking history, multiple measurements of different air pollution accumulation and biomarkers of oxidative damage were needed for further investigation.

## Conclusion

In conclusion, exposure to air pollutants significantly increases lung cancer risk, with an even greater risk for those genetically predisposed. This underscores the urgent necessity for governmental intervention aimed at enhancing air quality. Such measures are especially crucial as a means of prevention for those with heightened genetic susceptibility to lung cancer.

## Supplementary material

10.1136/bmjresp-2024-002899 online supplemental file 1

10.1136/bmjresp-2024-002899 online supplemental file 2

10.1136/bmjresp-2024-002899 online supplemental file 3

10.1136/bmjresp-2024-002899 online supplemental file 4

10.1136/bmjresp-2024-002899 online supplemental file 5

## Data Availability

All data relevant to the study are included in the article or uploaded as supplementary information.
