# Supplementary figures and images for: Greater lung cancer polygenic risk score in higher air pollution areas linked to greater rate of lung adenocarcinoma: a single-centre study in East Asia
Source: BMJ Open Respir Res. 2025 Oct 13;12(1):e002899. doi: 10.1136/bmjresp-2024-002899 (PMC12519728; doi:10.1136/bmjresp-2024-002899)

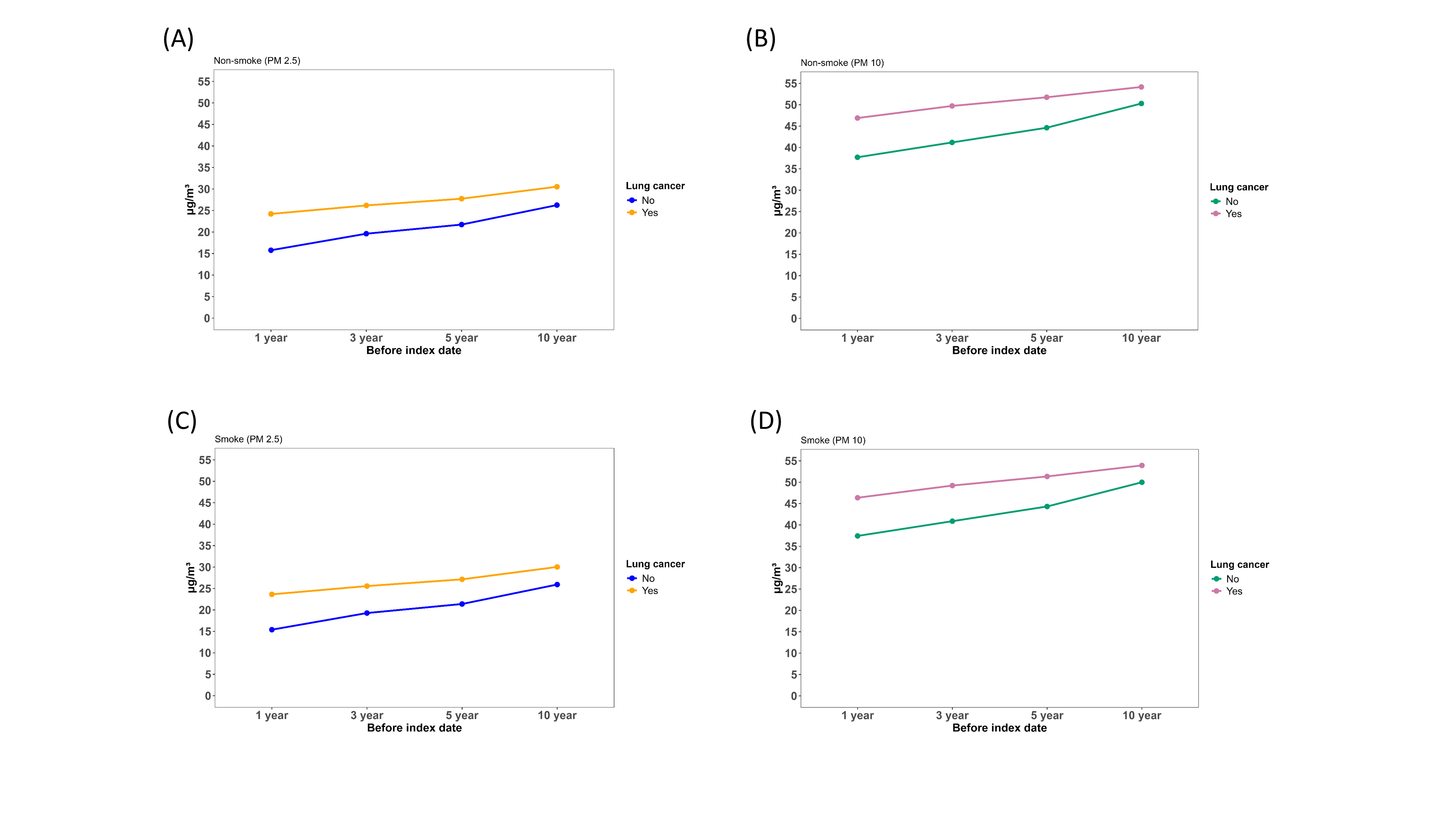

Supplement: online supplemental file 1 [file bmjresp-12-1-s001.tif]

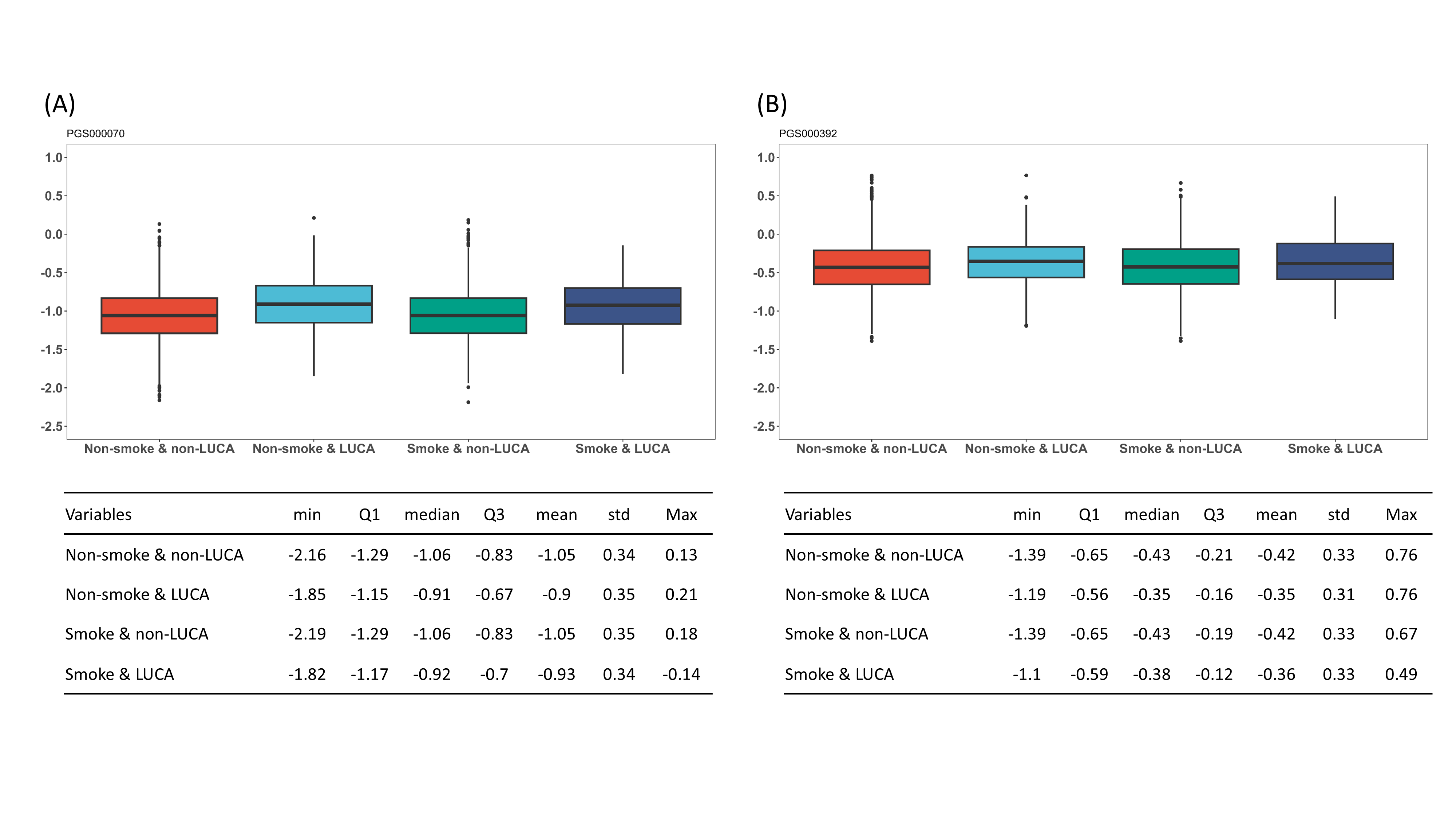

Supplement: online supplemental file 2 [file bmjresp-12-1-s002.tif]

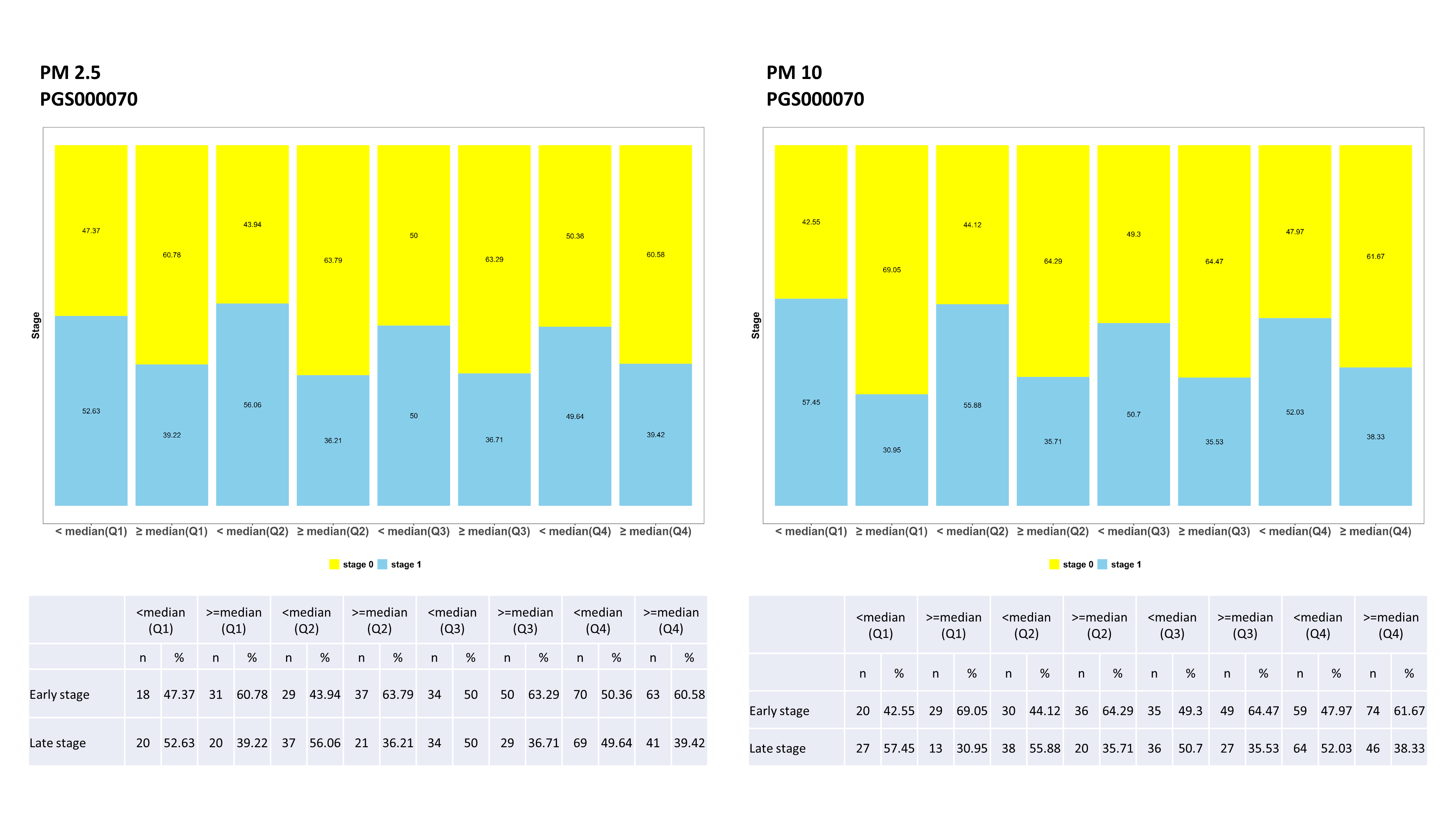

Supplement: online supplemental file 3 [file bmjresp-12-1-s003.TIF]

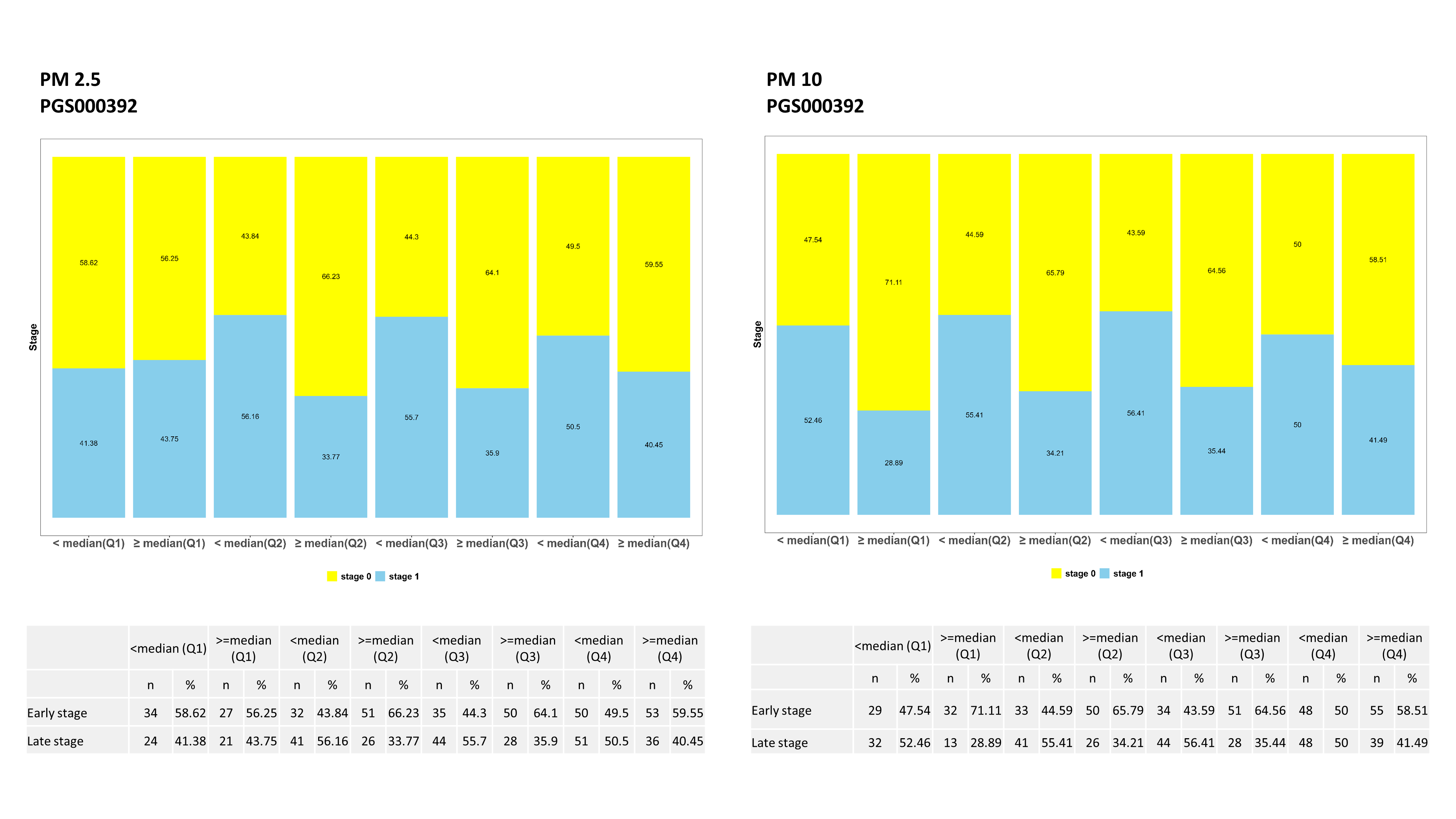

Supplement: online supplemental file 4 [file bmjresp-12-1-s004.TIF]
