# Supplementary material for: Greater lung cancer polygenic risk score in higher air pollution areas linked to greater rate of lung adenocarcinoma: a single-centre study in East Asia
Source: BMJ Open Respir Res. 2025 Oct 13;12(1):e002899. doi: 10.1136/bmjresp-2024-002899 (PMC12519728; doi:10.1136/bmjresp-2024-002899)
Supplement: online supplemental file 5 [file bmjresp-12-1-s005.docx]

**Supplementary Table 1**. ORs for LUCA in study population by various PRS groups.

| **PGS group** | **Reported Trait (PGS_Catalog)** | **Comparison** | **OR** | | **95% CI** | | ***P* value^a^** |
| --- | --- | --- | --- | --- | --- | --- | --- |
| PGS000070 | Lung cancer |  |  |  |  |  |  |
|  |  | Q4\|Q1 | 2.796 | | 2.236 | 3.497 | <0.0001 |
|  |  | Q3\|Q1 | 1.824 | | 1.438 | 2.313 | <0.0001 |
|  |  | Q2\|Q1 | 1.444 | | 1.125 | 1.852 | 0.0039 |
|  |  |  |  |  |  |  |  |
| PGS000789 | Lung cancer |  |  |  |  |  |  |
|  |  | Q4\|Q1 | 1.548 | | 1.264 | 1.896 | <0.0001 |
|  |  | Q3\|Q1 | 1.149 | | 0.926 | 1.427 | 0.2064 |
|  |  | Q2\|Q1 | 1.084 | | 0.871 | 1.35 | 0.4679 |
|  |  |  |  |  |  |  |  |
| PGS000078 | Lung cancer |  |  |  |  |  |  |
|  |  | Q4\|Q1 | 1.477 | | 1.202 | 1.816 | 0.0002 |
|  |  | Q3\|Q1 | 1.258 | | 1.017 | 1.557 | 0.0347 |
|  |  | Q2\|Q1 | 1.126 | | 0.905 | 1.402 | 0.2859 |
|  |  |  |  |  |  |  |  |
| PGS000389 | Cancer of bronchus; lung |  |  |  |  |  |  |
|  |  | Q4\|Q1 | 1.539 | | 1.243 | 1.905 | <0.0001 |
|  |  | Q3\|Q1 | 1.348 | | 1.083 | 1.677 | 0.0074 |
|  |  | Q2\|Q1 | 1.353 | | 1.088 | 1.684 | 0.0066 |
|  |  |  |  |  |  |  |  |
| PGS000390 | Cancer of bronchus; lung |  |  |  |  |  |  |
|  |  | Q4\|Q1 | 1.539 | | 1.243 | 1.905 | <0.0001 |
|  |  | Q3\|Q1 | 1.348 | | 1.083 | 1.677 | 0.0074 |
|  |  | Q2\|Q1 | 1.353 | | 1.088 | 1.684 | 0.0066 |
|  |  |  |  |  |  |  |  |
| PGS000391 | Cancer of bronchus; lung |  |  |  |  |  |  |
|  |  | Q4\|Q1 | 1.644 | | 1.326 | 2.038 | <0.0001 |
|  |  | Q3\|Q1 | 1.591 | | 1.284 | 1.972 | <0.0001 |
|  |  | Q2\|Q1 | 1.287 | | 1.027 | 1.614 | 0.0288 |
|  |  |  |  |  |  |  |  |
| PGS000392 | Cancer of bronchus; lung |  |  |  |  |  |  |
|  |  | Q4\|Q1 | 1.938 | | 1.559 | 2.409 | <0.0001 |
|  |  | Q3\|Q1 | 1.508 | | 1.202 | 1.891 | 0.0004 |
|  |  | Q2\|Q1 | 1.483 | | 1.181 | 1.862 | 0.0007 |
| PGS000880 | Lung cancer |  |  |  |  |  |  |
|  |  | Q4\|Q1 | 1.525 | | 1.246 | 1.866 | <0.0001 |
|  |  | Q3\|Q1 | 1.224 | | 0.991 | 1.511 | 0.0609 |
|  |  | Q2\|Q1 | 0.912 | | 0.727 | 1.144 | 0.426 |

^a^ Comparisons of categorical variables were analyzed using the univariable logistic regression.

**Supplementary Table 2.** **Comparison of long-term PM exposures (1-, 3-, 5-, and 10-year) by LUCA status and** **smoking status.**

**Lung cancer group**

| Variables | Non-smoke (n=458) | | Smoke (n=190) | | P-value |
| --- | --- | --- | --- | --- | --- |
| **Air pollution (Mean, SD) ^b^** |  |  |  |  |  |
| PM2.5 |  |  |  |  |  |
| 1 year before index date | 24.21 | 5.57 | 23.64 | 5.65 | 0.240 |
| 3 year before index date | 26.18 | 5.12 | 25.57 | 5.05 | 0.167 |
| 5 year before index date | 27.74 | 4.63 | 27.14 | 4.54 | 0.132 |
| 10 year before index date | 30.53 | 3.89 | 30.03 | 3.8 | 0.138 |
|  |  |  |  |  |  |
| PM10 |  |  |  |  |  |
| 1 year before index date | 46.89 | 6.69 | 46.37 | 6.65 | 0.364 |
| 3 year before index date | 49.72 | 5.63 | 49.22 | 5.56 | 0.301 |
| 5 year before index date | 51.75 | 4.6 | 51.35 | 4.54 | 0.312 |
| 10 year before index date | 54.16 | 3.36 | 53.93 | 3.23 | 0.415 |
| Using student T test. |  |  |  |  |  |

**Non-Lung cancer (control group)**

| Variables | Non-smoke (n=4944) | | Smoke (n=1514) | | P-value |
| --- | --- | --- | --- | --- | --- |
| **Air pollution (Mean, SD) ^b^** |  |  |  |  |  |
| PM2.5 |  |  |  |  |  |
| 1 year before index date | 15.77 | 2.74 | 15.4 | 2.53 | <0.0001 |
| 3 year before index date | 19.61 | 2.73 | 19.28 | 2.61 | <0.0001 |
| 5 year before index date | 21.73 | 2.79 | 21.39 | 2.69 | <0.0001 |
| 10 year before index date | 26.25 | 2.57 | 25.93 | 2.58 | <0.0001 |
|  |  |  |  |  |  |
| PM10 |  |  |  |  |  |
| 1 year before index date | 37.71 | 2.6 | 37.43 | 2.42 | 0.0001 |
| 3 year before index date | 41.18 | 2.58 | 40.88 | 2.45 | <0.0001 |
| 5 year before index date | 44.62 | 2.47 | 44.32 | 2.37 | <0.0001 |
| 10 year before index date | 50.29 | 2.44 | 49.99 | 2.49 | <0.0001 |

Using student T test.

**Supplementary Table 3.** Risk of PRSs, smoke and air pollution in the participants.

| **Variables** | | **Risk of LUCA** | | |  |
| --- | --- | --- | --- | --- | --- |
|  |  |  |  |  |  |
|  |  | **OR** | **95% CI** | ***P* value^a^** |  |
| **PGS000070** | |  |  |  |  |
|  | Q1 | - | - | - |  |
|  | Q4 | 2.913 | 2.270-3.739 | <0.0001 |  |
| Smoke |  |  |  |  |  |
|  | no | - | - | - |  |
|  | yes | 1.391 | 1.089-1.777 | 0.0083 |  |
|  |  |  |  |  |  |
|  | | **Risk of LUCA** | | |  |
|  |  |  |  |  |  |
|  |  | **OR** | **95% CI** | ***P* value^a^** |  |
| **PGS000392** | |  |  |  |  |
|  | Q1 | - | - | - |  |
|  | Q4 | 1.98 | 1.552-2.526 | <0.0001 |  |
| Smoke |  |  |  |  |  |
|  | no | - | - | - |  |
|  | yes | 1.461 | 1.136-1.881 | 0.0032 |  |

^a^ Comparisons of categorical variables were analyzed using the univariable logistic regression, adjusted for age and gender.

**Supplementary Table 4**. Association of PGS000070 with lung cancer risk stratified by smoking status and particulate matter exposures (PM2.5, PM10).

| **Variables** | **Current-Smoking** | | | |  | **Current-Smoking** | | | |
| --- | --- | --- | --- | --- | --- | --- | --- | --- | --- |
|  | **PM2.5<median** | | | |  | **PM2.5>=median** | | | |
|  | **OR** | **95% CI** | | ***P* value** |  | **OR** | **95% CI** | | ***P* value^a^** |
| **PGS000070** | |  |  |  |  |  |  |  |  |
| Q1 | - | - | - | - |  | - | - | - | - |
| Q4 | >999 | <0.001 | >999 | 0.9403 |  | 5.625 | 1.701 | 18.606 | 0.0047 |
|  |  |  |  |  |  |  |  |  |  |
|  | **Former-smoking** | | | |  | **Former-smoking** | | | |
|  | **PM2.5<median** | | | |  | **PM2.5>=median** | | | |
|  | **OR** | **95% CI** | | ***P* value** |  | **OR** | **95% CI** | | ***P* value^a^** |
| Q1 | - | - | - | - |  | - | - | - | - |
| Q4 | 0.328 | 0.061 | 1.763 | 0.1938 |  | 3.672 | 2.003 | 6.73 | <0.0001 |
|  |  |  |  |  |  |  |  |  |  |
|  | **Non-smoking** | | | |  | **Non-smoking** | | | |
|  | **PM2.5<median** | | | |  | **PM2.5>=median** | | | |
|  | **OR** | **95% CI** | | ***P* value** |  | **OR** | **95% CI** | | ***P* value^a^** |
| Q1 | - | - | - | - |  | - | - | - | - |
| Q4 | 2.169 | 0.539 | 8.724 | 0.2757 |  | 3.01 | 2.207 | 4.106 | <0.0001 |
|  |  |  |  |  |  |  |  |  |  |
|  | **Current-Smoking** | | | |  | **Current-Smoking** | | | |
|  | **PM10<median** | | | |  | **PM10>=median** | | | |
|  | **OR** | **95% CI** | | ***P* value** |  | **OR** | **95% CI** | | ***P* value^a^** |
| Q1 | - | - | - | - |  | - | - | - | - |
| Q4 | >999 | <0.001 | >999 | 0.944 |  | 5.331 | 1.626 | 17.485 | 0.0058 |
|  |  |  |  |  |  |  |  |  |  |
|  | **Former-smoking** | | | |  | **Former-smoking** | | | |
|  | **PM10<median** | | | |  | **PM10>=median** | | | |
|  | **OR** | **95% CI** | | ***P* value** |  | **OR** | **95% CI** | | ***P* value^a^** |
| Q1 | - | - | - | - |  | - | - | - | - |
| Q4 | 1.938 | 0.453 | 8.292 | 0.3723 |  | 2.358 | 1.305 | 4.26 | 0.0045 |
|  |  |  |  |  |  |  |  |  |  |
|  | **Non-smoking** | | | |  | **Non-smoking** | | | |
|  | **PM10<median** | | | |  | **PM10>=median** | | | |
|  | **OR** | **95% CI** | | ***P* value** |  | **OR** | **95% CI** | | ***P* value^a^** |
| Q1 | - | - | - | - |  | - | - | - | - |
| Q4 | 2.585 | 0.986 | 6.779 | 0.0536 |  | 2.85 | 2.076 | 3.913 | <0.0001 |

^a^ ORs were estimated using univariable logistic regression models, adjusted for age and gender.

**Supplementary Table 5**. Association of PGS000392 with lung cancer risk stratified by smoking status and particulate matter exposures (PM2.5, PM10).

| **Variables** | **Current-Smoking** | | | |  | **Current-Smoking** | | | |
| --- | --- | --- | --- | --- | --- | --- | --- | --- | --- |
|  | **PM2.5<median** | | | |  | **PM2.5>=median** | | | |
|  | **OR** | **95% CI** | | ***P* value** |  | **OR** | **95% CI** | | ***P* value^a^** |
| **PGS000392** | |  |  |  |  |  |  |  |  |
| Q1 | - | - | - | - |  | - | - | - | - |
| Q4 | >999 | <0.001 | >999 | 0.9564 |  | 2.206 | 0.709 | 6.858 | 0.1717 |
|  |  |  |  |  |  |  |  |  |  |
|  | **Former-smoking** | | | |  | **Former-smoking** | | | |
|  | **PM2.5<median** | | | |  | **PM2.5>=median** | | | |
|  | **OR** | **95% CI** | | ***P* value** |  | **OR** | **95% CI** | | ***P* value^a^** |
| Q1 | - | - | - | - |  | - | - | - | - |
| Q4 | 1.12 | 0.203 | 6.161 | 0.8966 |  | 1.564 | 0.912 | 2.682 | 0.1037 |
|  |  |  |  |  |  |  |  |  |  |
|  | **Non-smoking** | | | |  | **Non-smoking** | | | |
|  | **PM2.5<median** | | | |  | **PM2.5>=median** | | | |
|  | **OR** | **95% CI** | | ***P* value** |  | **OR** | **95% CI** | | ***P* value^a^** |
| Q1 | - | - | - | - |  | - | - | - | - |
| Q4 | 1.081 | 0.311 | 3.753 | 0.9026 |  | 2.199 | 1.61 | 3.003 | <0.0001 |
|  |  |  |  |  |  |  |  |  |  |
|  | **Current-Smoking** | | | |  | **Current-Smoking** | | | |
|  | **PM10<median** | | | |  | **PM10>=median** | | | |
|  | **OR** | **95% CI** | | ***P* value** |  | **OR** | **95% CI** | | ***P* value^a^** |
| Q1 | - | - | - | - |  | - | - | - | - |
| Q4 | 2.713 | 0.272 | 27.075 | 0.3952 |  | 2.541 | 0.728 | 8.877 | 0.1438 |
|  |  |  |  |  |  |  |  |  |  |
|  | **Former-smoking** | | | |  | **Former-smoking** | | | |
|  | **PM10<median** | | | |  | **PM10>=median** | | | |
|  | **OR** | **95% CI** | | ***P* value** |  | **OR** | **95% CI** | | ***P* value^a^** |
| Q1 | - | - | - | - |  | - | - | - | - |
| Q4 | 3.162 | 0.317 | 31.581 | 0.3269 |  | 1.37 | 0.804 | 2.334 | 0.2467 |
|  |  |  |  |  |  |  |  |  |  |
|  | **Non-smoking** | | | |  | **Non-smoking** | | | |
|  | **PM10<median** | | | |  | **PM10>=median** | | | |
|  | **OR** | **95% CI** | | ***P* value** |  | **OR** | **95% CI** | | ***P* value^a^** |
| Q1 | - | - | - | - |  | - | - | - | - |
| Q4 | 1.248 | 0.45 | 3.461 | 0.6708 |  | 2.183 | 1.593 | 2.991 | <0.0001 |

^a^ ORs were estimated using univariable logistic regression models, adjusted for age and gender.

**Supplementary Table 6-1.** Interaction effect of PRS and PM₂.₅/PM₁₀ on LUCA risk (PGS000070).

| **Multiplicative interaction** | **OR** | **95% CI** | ***p* value** |  | **Multiplicative interaction** | **OR** | **95% CI** | ***p* value** |
| --- | --- | --- | --- | --- | --- | --- | --- | --- |
| Sex (Male) | 1.04 | 0.88 to 1.24 | 0.63 |  | Sex (Male) | 1.06 | 0.89 to 1.26 | 0.54 |
| Age | 1 | 1.00 to 1.01 | 0.22 |  | Age | 1 | 0.99 to 1.01 | 0.75 |
| PM2.5 (> median) | 10.66 | 5.33 to 21.32 | <0.0001 |  | PM10 (> median) | 11.61 | 5.81 to 23.23 | <0.0001 |
| PGS000070 (Q2) | 0.76 | 0.28 to 2.05 | 0.589 |  | PGS000070 (Q2) | 1.26 | 0.52 to 3.06 | 0.61 |
| PGS000070 (Q3) | 0.77 | 0.29 to 2.07 | 0.6 |  | PGS000070 (Q3) | 1.41 | 0.59 to 3.36 | 0.44 |
| PGS000070 (Q4) | 1.28 | 0.53 to 3.09 | 0.59 |  | PGS000070 (Q4) | 2.72 | 1.25 to 5.94 | 0.01 |
| PM2.5*PGS00070 (Q2) | 2.15 | 0.76 to 6.03 | 0.15 |  | PM10*PGS00070 (Q2) | 1.18 | 0.46 to 2.99 | 0.73 |
| PM2.5*PGS00070 (Q3) | 2.62 | 0.94 to 7.36 | 0.07 |  | PM10*PGS00070 (Q3) | 1.28 | 0.51 to 3.19 | 0.6 |
| PM2.5*PGS00070 (Q4) | 2.52 | 1.00 to 6.36 | 0.051 |  | PM10*PGS00070 (Q4) | 1.04 | 0.46 to 2.38 | 0.92 |
| **Additive interaction** | **Estimates** | **95% CI** |  |  | **Additive interaction** | **Estimates** | **95% CI** |  |
| Multiplicative | 0.77 | 0.27 to 2.07 |  |  | Multiplicative | 1.41 | 0.59 to 3.47 |  |
| RERI | -4.19 | -12.05 to 3.68 |  |  | RERI | 8.75 | -27.44 to 44.95 |  |
| AP | -0.67 | -3.36 to 2.02 |  |  | AP | 0.42 | -0.43 to 1.28 |  |
| SI | 0.55 | 0.08 to 3.97 |  |  | SI | 1.81 | 0.39 to 8.33 |  |

Adjusted for age, sex, air pollution, and quartile of PRS.

The PRS deciles were determined based on both lung cancer cases and controls. High air pollution: 51% - 100% (≥16.49 ug/m3); low air pollution: 0-50% (≤12.78 ug/m3).

**Supplementary Table 6-2.** Interaction effect of PRS and PM₂.₅/PM₁₀ on LUCA risk (PGS000392).

| **Multiplicative interaction** | **OR** | **95% CI** | ***p* value** |  | **Multiplicative interaction** | **OR** | **95% CI** | ***p* value** |
| --- | --- | --- | --- | --- | --- | --- | --- | --- |
| Sex (Male) | 1.04 | 0.87 to 1.24 | 0.67 |  | Sex (Male) | 1.06 | 0.89 to 1.26 | 0.53 |
| Age | 1 | 1.00 to 1.01 | 0.28 |  | Age | 1 | 0.99 to 1.01 | 0.83 |
| PM2.5 (> median) | 16.05 | 7.77 to 33.15 | <0.0001 |  | PM10 (> median) | 13.51 | 6.79 to 26.86 | <0.0001 |
| PGS000392 (Q2) | 1.29 | 0.51 to 3.29 | 0.59 |  | PGS000392 (Q2) | 1.99 | 0.89 to 4.45 | 0.09 |
| PGS000392 (Q3) | 0.98 | 0.35 to 2.72 | 0.97 |  | PGS000392 (Q3) | 1.58 | 0.67 to 3.71 | 0.3 |
| PGS000392 (Q4) | 1.2 | 0.46 to 3.13 | 0.71 |  | PGS000392 (Q4) | 1.68 | 0.72 to 3.90 | 0.23 |
| PM2.5*PGS000392 (Q2) | 1.21 | 0.46 to 3.21 | 0.7 |  | PM10*PGS000392 (Q2) | 0.88 | 0.34 to 1.86 | 0.6 |
| PM2.5*PGS000392 (Q3) | 1.55 | 0.54 to 4.45 | 0.41 |  | PM10*PGS000392 (Q3) | 0.97 | 0.40 to 2.37 | 0.94 |
| PM2.5*PGS000392 (Q4) | 1.68 | 0.62 to 4.51 | 0.31 |  | PM10*PGS000392 (Q4) | 1.17 | 0.48 to 2.82 | 0.73 |
| **Additive interaction** | **Estimates** | **95% CI** |  |  | **Additive interaction** | **Estimates** | **95% CI** |  |
| Multiplicative | 0.98 | 0.34 to 2.75 |  |  | Multiplicative | 1.58 | 0.68 to 3.84 |  |
| RERI | 4.04 | -32.04 to 40.14 |  |  | RERI | 27.86 | -51.46 to 107.18 |  |
| AP | 0.2 | -1.12 to 1.52 |  |  | AP | 0.66 | 0.17 to 1.15 |  |
| SI | 1.26 | 0.23 7.06 |  |  | SI | 3.06 | 0.72 to 12.95 |  |

Adjusted for age, sex, air pollution, and quartile of PRS.

The PRS deciles were determined based on both lung cancer cases and controls. High air pollution: 51% - 100% (≥16.49 ug/m3); low air pollution: 0-50% (≤12.78 ug/m3).

RERI, relative excess risk due to interaction; AP, attributable proportion due to interaction; SI, synergy index; OR, odd ratio. The 95% confidence interval (95% CI) of the interaction indicators was calculated using the table produced by T Anderson. If the confidence NaN, not a number. interval of RERI or AP contains 0 or the confidence interval of SI contains 1, it indicates that the two factors have no interaction.
